# Age and intraoperative cartilage status as determinants of mid‐term functional outcomes after hip arthroscopy

**DOI:** 10.1002/jeo2.70806

**Published:** 2026-06-15

**Authors:** Andrea Burla, Irene Tampieri, Luca Vigliaroli, Valentina Fantoni, Francesco Aparo, Margherita Bonaiuti, Eleonora Olivotto, Stefano Zaffagnini, Enrico Tassinari

**Affiliations:** ^1^ Department of Biomedical and Neuromotor Sciences DIBINEM Alma Mater Studiorum University of Bologna Bologna Italy; ^2^ 2nd Orthopaedic and Trauma Clinic, IRCCS Rizzoli Orthopaedic Institute Bologna Italy; ^3^ RAMSES Laboratory, RIT Department, Research Centre Codivilla‐Putti, IRCCS Rizzoli Orthopaedic Institute Bologna Italy

**Keywords:** age, femoroacetabular impingement, hip arthroscopy, patient‐reported outcome measures, prognostic factors

## Abstract

**Purpose:**

The purpose of this study was to evaluate mid‐term functional outcomes after hip arthroscopy for femoroacetabular impingement syndrome and to identify prognostic factors associated with postoperative outcomes at a minimum follow‐up of 5 years.

**Methods:**

A retrospective analysis was conducted on patients who underwent hip arthroscopy for femoroacetabular impingement syndrome performed by a single surgeon between 2015 and 2020. Patient‐reported outcome measures (PROMs), including the Hip Outcome Score, Hip Outcome Score Sports Subscale, Harris Hip Score and Western Ontario and McMaster Universities Osteoarthritis Index, were collected preoperatively and at a minimum follow‐up of 5 years. Intraoperative femoral and acetabular cartilage status was graded according to the Outerbridge classification. Univariate linear regression analyses were performed to identify variables associated with postoperative outcomes. Multivariate linear regression analysis was performed only for outcomes showing more than one candidate predictor at univariate analysis.

**Results:**

A total of 87 patients, accounting for 91 hips, were included. All PROMs demonstrated significant improvement from preoperative to postoperative assessment: Hip Outcome Score improved from 68.3 ± 8.5 to 93.5 ± 8.7, Hip Outcome Score Sports Subscale from 68.1 ± 9.0 to 92.4 ± 10.6, Harris Hip Score from 74.1 ± 9.9 to 95.4 ± 7.0 and Western Ontario and McMaster Universities Osteoarthritis Index from 14.5 ± 5.9 to 2.7 ± 5.7 (all *p* < 0.001). Increasing age was significantly associated with worse postoperative Hip Outcome Score (*p* < 0.001), Hip Outcome Score Sports Subscale (*p* = 0.037), and Harris Hip Score (*p* = 0.004), as well as with higher Western Ontario and McMaster Universities Osteoarthritis Index scores (*p* = 0.013). No significant associations were found between postoperative outcomes and sex, body mass index, labral pathology, or femoral and acetabular cartilage status. Multivariate analysis identified age as the only independent predictor of postoperative Harris Hip Score (*β* = −0.22; *p* = 0.005).

**Conclusions:**

At mid‐term follow‐up after hip arthroscopy, patient age was identified as an independent predictor of functional outcomes, whereas intraoperative cartilage status was not independently associated with postoperative outcomes. These findings suggest that age should be considered a key prognostic factor in patient selection and preoperative counseling for hip arthroscopy.

**Level of Evidence:**

Level IV.

**Abbreviations**
BMI: body mass index
FAI: femoroacetabular impingement
FAIS: femoroacetabular impingement syndrome
HHS: Harris Hip Score
HOS: Hip Outcome Score
HOS‐SS: Hip Outcome Score – Sports Subscale
LCEA: lateral centre‐edge angle
PROMs: patient‐reported outcome measures
WOMAC: Western Ontario and McMaster Universities Osteoarthritis Index

## INTRODUCTION

Hip pain is a common complaint among patients with high functional demands, such as athletes [4, 17, 24]. Among young individuals, one of the primary etiologies is femoroacetabular impingement syndrome (FAIS). FAIS is characterised by early abnormal mechanical conflict between the proximal femur and the acetabular rim, resulting in reduced passive internal rotation of the hip. This condition arises from an anatomical deformity—acetabular, femoral, or a combination of both—that leads to abnormal contact during hip motion and repetitive activities of daily living and sports. These repetitive microtraumas frequently result in chondral damage involving both the femoral head and the acetabular surface [11]. The acetabular labrum, a key structure for hip stability and joint sealing, is also commonly affected [18].

Contemporary evidence demonstrates that surgical management of FAIS provides superior outcomes compared with conservative treatment. Hip arthroscopy has become the preferred technique for addressing labral pathology and correcting underlying bony abnormalities in a minimally invasive manner [3].

Recent studies have further emphasised that outcomes after hip arthroscopy are influenced by multiple factors, including surgical technique, capsular management, patient‐specific predictive variables and acetabular morphology, particularly in patients with borderline dysplasia [1, 14, 19, 23].

Despite its favourable profile, hip arthroscopy is not devoid of complications and postoperative outcomes may be influenced by several prognostic factors [13]. Previous studies have evaluated the impact of body mass index (BMI), sex, age and preoperative cartilage status on clinical results and return to sports. Cartilage integrity, in particular, is a critical determinant of long‐term benefit [6, 16]. Recent evidence has further highlighted the prognostic relevance of intraoperative cartilage status after hip arthroscopy, with worse outcomes reported in patients with more advanced chondral damage. At the same time, the role of hip arthroscopy in the presence of these degenerative intra‐articular changes remains an area of ongoing clinical equipoise [2, 15].

However, although several studies have investigated predictors of outcome after hip arthroscopy, most reports focus on short‐term follow‐up, and data regarding mid‐term outcomes remain comparatively limited. In addition, the relative contribution of patient age compared with intra‐operative cartilage findings remains debated.

Given these considerations, identifying the factors most strongly associated with clinical outcomes is essential to optimise patient selection and provide appropriate surgical indications.

The purpose of this retrospective study was to evaluate the mid‐term outcomes of primary hip arthroscopy with a minimum follow‐up of 5 years and to analyse the prognostic variables most strongly associated with the recovery of daily and sports activities.

## MATERIALS AND METHODS

### Patient selection

This study was conducted and reported according to the STROBE (Strengthening the Reporting of Observational Studies in Epidemiology) guidelines for observational cohort studies [22].

Ethical approval was obtained from the Local Ethical Committee of IRCCS Istituto Ortopedico Rizzoli, Bologna, Italy (Protocol No. 0012125). All procedures were conducted in accordance with the Declaration of Helsinki, and all participants provided written informed consent.

Patients who underwent hip arthroscopy for FAIS between 1 January 2015 and 31 December 2020 were retrospectively identified. All procedures were performed at IRCCS Istituto Ortopedico Rizzoli by a single surgeon. The study cohort was derived from a consecutive surgical series performed during the study period.

Between 2015 and 2020, a total of 161 hip arthroscopies for FAIS were performed. After applying the inclusion criteria and the requirement of a minimum follow‐up of 5 years, 87 patients (91 hips) were available for final analysis. Eighteen hips were excluded because they underwent total hip arthroplasty (THA) before reaching the minimum follow‐up, while the remaining non‐included patients were excluded because they did not respond or declined to participate in the study.

Eligible patients were aged between 15 and 55 years at the time of surgery and underwent primary hip arthroscopy. The lower age limit was selected to include skeletally mature patients, whereas the upper age limit was chosen to reduce the potential confounding effect of advanced age‐related degenerative changes on postoperative outcomes and to focus the analysis on the population most commonly considered for primary hip arthroscopy for FAIS. Exclusion criteria included revision hip arthroscopy, any surgical procedure on the ipsilateral lower limb within the previous 12 months, inflammatory or rheumatic diseases, uncontrolled metabolic disorders and follow‐up shorter than 5 years. No exclusion criterion based on Tönnis grade was applied.

The primary outcome measure was the Hip Outcome Score (HOS), including both the Activities of Daily Living (HOS‐ADL) and Sports Subscale (HOS‐SS). Secondary outcome measures included the Harris Hip Score (HHS) and the Western Ontario and McMaster Universities Osteoarthritis Index (WOMAC).

### Preoperative evaluation and radiographic analysis

All patients underwent a standardised preoperative clinical evaluation including collection of demographic data (age, sex, weight, BMI and comorbidities) and physical examination of hip function.

Standard radiographs included an anteroposterior pelvis view and a Dunn lateral view of the symptomatic hip. Radiographic measurements included the lateral centre‐edge angle (LCEA), Tönnis angle and α‐angle. Degenerative changes were graded according to the Tönnis classification. Tönnis grade was recorded for all patients as part of the preoperative radiographic assessment.

Magnetic resonance imaging (MRI) was performed in all cases to evaluate cartilage integrity and detect acetabular labral pathology.

In cases of diagnostic uncertainty, an ultrasound‐guided intra‐articular injection of local anaesthetic was used as a diagnostic test. A positive response was defined as a marked reduction in hip pain, particularly during movements that usually reproduced symptoms, within a few minutes after injection.

All patients underwent at least 10 weeks of conservative treatment prior to surgery, consisting of nonsteroidal anti‐inflammatory drugs, physiotherapy and activity modification.

### Surgical technique

Patients were positioned on a traction table under general anaesthesia. Arthroscopy was performed using standard anterolateral and mid‐anterior portals with an interportal capsulotomy.

A diagnostic arthroscopy was first performed to assess cartilage status and identify chondral lesions, which were graded according to the Outerbridge classification. The acetabular labrum was evaluated for the presence and type of tears.

Labral treatment consisted of debridement, thermocoagulation or suture repair depending on the characteristics of the lesion. Full‐thickness acetabular chondral lesions (Outerbridge grade IV) were treated with microfracture when indicated.

**Figure 1:**
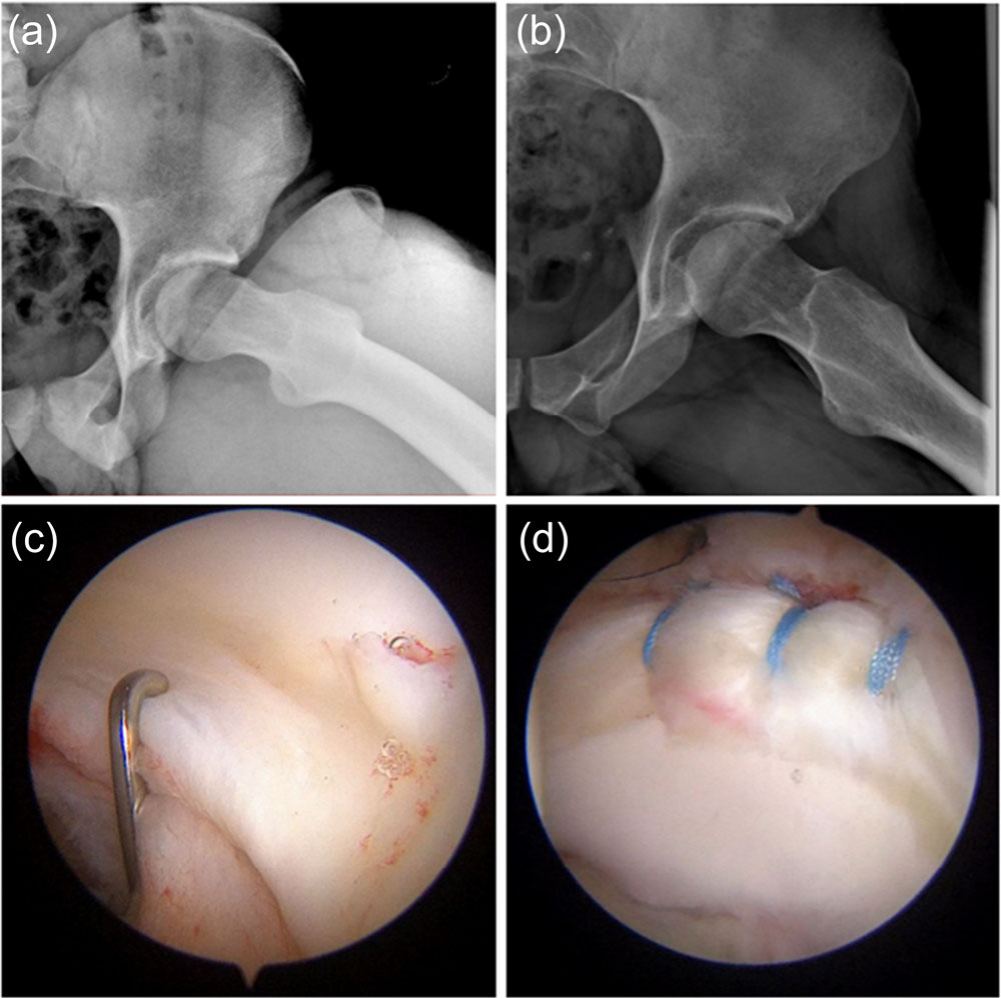
Representative radiographic and arthroscopic findings in a patient undergoing hip arthroscopy for femoroacetabular impingement. (a) Preoperative Dunn view showing cam‐type deformity. (b) Postoperative Dunn view after femoral osteoplasty. (c) Arthroscopic view of acetabular cartilage lesion. (d) Arthroscopic view following labral repair.

A representative case illustrating preoperative radiographic findings, intraoperative cartilage lesions, and postoperative correction is shown in Figure 1.

### Post‐op rehabilitation protocol

All patients followed a standardised rehabilitation protocol. Partial weight‐bearing with two crutches was allowed for 4 weeks. Hip motion was permitted immediately after surgery with flexion limited to 0°–90° and avoidance of extreme rotation and adduction.

Patients undergoing microfracture were instructed to maintain toe‐touch weight‐bearing for 4 weeks.

All patients received prophylaxis against heterotopic ossification with celecoxib 200 mg daily for 4 weeks.

### Statistical Analysis

Descriptive statistics, including means with standard deviations for continuous variables and proportions for categorical variables, were used to summarise patient characteristics. Normality of quantitative variables was assessed using the Shapiro–Wilk test.

The comparison of pre‐ and post‐operative patient‐reported outcome measures (PROMs: HOS, HOS‐SS, HHS and WOMAC) was obtained using a paired Wilcoxon signed‐rank.

To evaluate the association between clinical variables and postoperative PROMs, univariate linear regression analyses were performed. The dependent variable was defined as PROMs while the independent variables were age, sex, BMI, acetabular labral tear, femoral chondropathy, acetabular chondropathy, focal cartilage lesion (grade IV).

Femoral and acetabular chondropathy were classified according to the Outerbridge classification and then grouped as mild (grades 0–1) or severe (grades 2–3) for regression analysis.

To limit the number of predictor variables in relation to the number of observations, variables were selected for multivariable modelling based on trends identified in the univariate linear regression analyses (*p* < 0.10) [10].

Multivariable regression analysis was performed only when more than one candidate predictor was identified in the univariate analysis, in order to reduce the risk of model overfitting given the available sample size.

A *p*‐value < 0.05 was considered statistically significant. All analyses were performed using JASP (version 0.95.1; JASP Team, University of Amsterdam, Netherlands).

An a priori power analysis was conducted using GPower software (Version 3.1.9.4, University of Düsseldorf, Germany) to determine the minimum sample size required for the primary outcome analysis. The sample size was calculated through an a priori power analysis based on the primary outcome of the study, namely the HOS score. A study in the literature with a rationale similar to the present project [16] as used as a reference. From that study, a standard deviation ranging between 14.8 and 27.1 points was extracted for the two components of the HOS score. From a conservative perspective, a standard deviation of 28 points was selected. Similarly, the difference between the means was assumed to be lower than the minimum standard deviation reported in the same study by a factor of 2/3, namely 10 points. This resulted in an effect size of 0.36 (considered ‘medium’), which was used for the power analysis, assuming a two‐tailed paired *t*‐test with *α* = 0.05 and a sample power (1–β) of 0.9. The minimum required sample size was therefore estimated at 85 patients. This power analysis referred to the primary preoperative‐to‐postoperative comparison and does not specifically establish power for the multivariable regression models; this aspect has therefore been acknowledged as a limitation of the study.

## RESULTS

A total of 87 patients were included; four patients underwent bilateral hip arthroscopy, resulting in 91 procedures. Considering procedures, there was a predominance of male hips (61; 67.0%) compared with female hips (30; 33.0%), with a mean age of 35.3 ± 9.2 years. The most frequently observed FAI morphology was cam‐type (60; 65.9%), while acetabular labral tears were present in the majority of cases (82; 90.1%). With regard to preoperative radiographic osteoarthritis, Tönnis grade 0 was observed in 34 hips (37.4%), grade 1 in 48 hips (52.7%), grade 2 in 7 hips (7.7%) and grade 3 in 2 hips (2.2%). Regarding cartilage status, femoral cartilage status according to the Outerbridge classification was grade 2–3 in 55 hips (60.5%), whereas acetabular cartilage status was grade 2–3 in 64 hips (70.4%). Focal full‐thickness (grade IV) cartilage lesions were observed in 26 patients (28.6%). Demographic, radiographic and intraoperative characteristics of the study cohort are summarised in Table 1.

**Table 1:** Demographic, radiographic and intraoperative characteristics of the study cohort.

Number of hip arthroscopies	91
Sex	
− Female	30 (33.0)
− Male	61 (67.0)
Age (years)	35.3 ± 9.2 [16.0; 53.0]
BMI (kg/m^2^)	24.2 ± 3.6 [17.8; 37.1]
Follow‐up (months)	84.6 ± 19.8 [56.0; 129.0]
Side	
− Left	34 (37.4)
− Right	57 (62.6)
FAI	
− Cam‐morphology	60 (65.9)
− Pincer‐morphology	2 (2.2)
− Mixed‐morphology	29 (31.9)
Acetabular labral tear	
− Yes	82 (90.1)
− No	9 (9.9)
Tonnis grade	
− 0 (normal)	34 (37.4)
− 1 (mild OA)	48 (57.2)
− 2 (moderate OA)	7 (7.7)
− 3 (severe OA)	2 (2.2)
Femoral cartilage status (Outerbridge)	
− 0	12 (13.2)
− 1	24 (26.4)
− 2	40 (44.0)
− 3	15 (16.5)
Acetabular cartilage status (Outerbridge)	
− 0	2 (2.2)
− 1	25 (27.5)
− 2	34 (37.4)
− 3	30 (33.0)
Focal cartilage lesion (grade IV)	
− Yes	26 (28.6)
− No	65 (71.4)

*Note*: Data are presented as mean ± standard deviation [min; max] or *n* (%). Tönnis grade refers to preoperative radiographic osteoarthritis, whereas femoral and acetabular cartilage status were graded intraoperatively according to the Outerbridge classification.

Abbreviations: BMI, body mass index; FAI, femoroacetabular impingement; OA, osteoarthritis.

**Figure 2:**
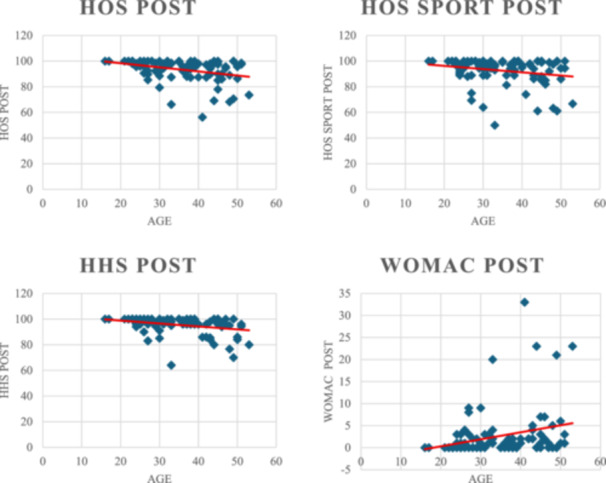
Scatter plots illustrating the relationship between age and postoperative patient‐reported outcome measures. HHS, Harris Hip Score; HOS, Hip Outcome Score; HOS‐SS, Hip Outcome Score – Sports Specific; WOMAC, Western Ontario and McMaster Universities Osteoarthritis Index.

Age showed a weak negative association with HOS Post, HOS Sport Post and HHS scores, while WOMAC scores tended to increase slightly with age (Figure 2).

**Figure 3:**
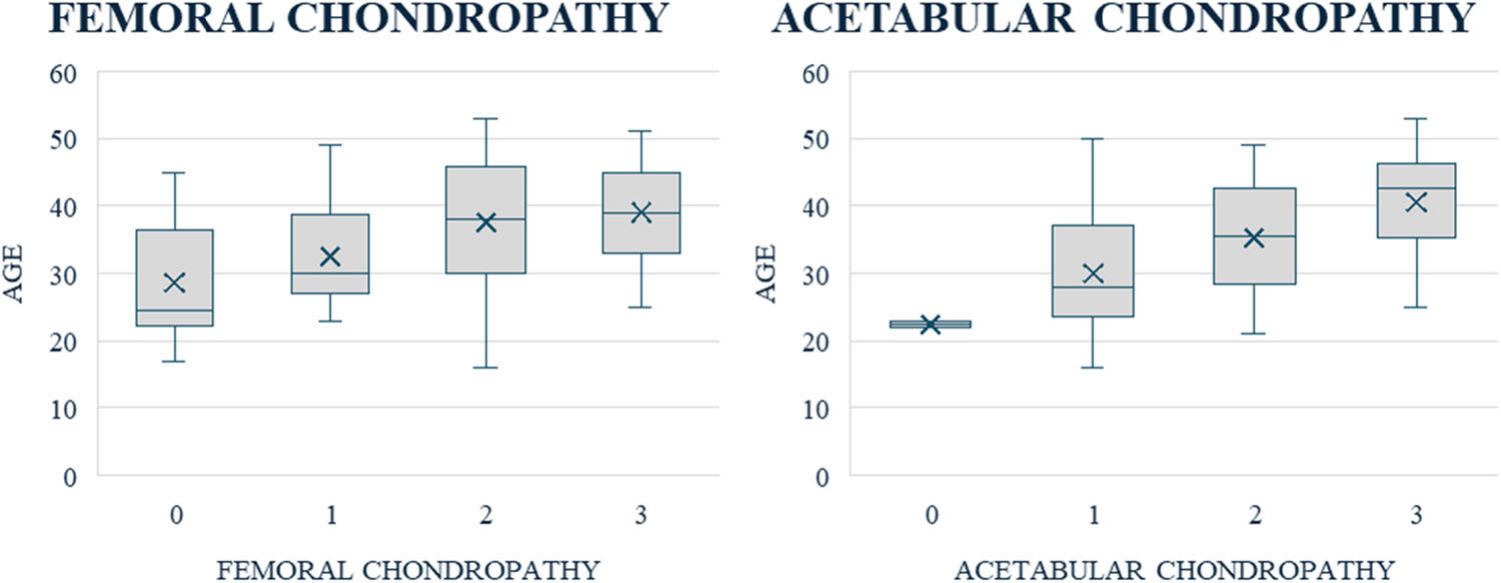
Boxplots illustrating the distribution of age according to the grades of femoral and acetabular chondropathy. The boxes represent the interquartile range (IQR), the horizontal line inside each box indicates the median, and the crosses indicate the mean age for each grade. Whiskers show the minimum and maximum values excluding outliers.

Age showed a progressive increase with higher grades of both femoral and acetabular cartilage status according to the Outerbridge classification. Patients with more advanced cartilage damage (grades 2 and 3) were generally older compared to those with lower grades (0 and 1) (Figure 3).

### Clinical scores

All PROMs demonstrated a statistically significant improvement from the pre‐operative to the post‐operative assessment (all *p* < 0.001). The HOS, HOS–sports subscale and HHS scores showed marked increases at follow‐up, whereas the WOMAC score significantly decreased, indicating reduced symptoms and improved function. The magnitude of change was large across all measures, as reflected by very strong rank‐biserial correlation values, suggesting a robust treatment effect (Table 2).

**Table 2:** Comparison of preoperative and postoperative patient‐reported outcome measures.

	Pre‐operative	Post‐operative	*p*‐Value	Rank‐Biserial correlation
HOS	68.3 ± 8.5	93.5 ± 8.7	<0.001	1.00
HOS‐SS	68.1 ± 9.0	92.4 ± 10.6	<0.001	0.98
HHS	74.1 ± 9.9	95.4 ± 7.0	<0.001	0.99
WOMAC	14.5 ± 5.9	2.7 ± 5.7	<0.001	0.98

*Note*: Data were expressed as mean ± standard deviation [min; max]. The reported *p*‐values were obtained using a Wilcoxon signed‐rank to assess the differences between time (pre vs. post).

Abbreviations: HHS, Harris Hip Score; HOS, Hip Outcome Score; HOS‐SS, Hip Outcome Score – Sports Specific; WOMAC, Western Ontario and McMaster Universities Osteoarthritis Index.

As reported in Table 3, the univariate regression analysis identified potential associations (*p* < 0.10) mainly for age. Increasing age was associated with lower post‐operative HOS (*p* < 0.001), HOS‐SS (*p* = 0.037) and HHS (*p* = 0.004) scores, while a positive association with higher WOMAC scores was observed (*p* = 0.013). In addition, the presence of an acetabular labral tear showed a trend toward worse post‐operative HHS scores (*p* = 0.083). No other variables demonstrated relevant associations with post‐operative PROMs (*p* ≥ 0.10).

**Table 3:** Univariate regression analysis for postoperative patient‐reported outcome measures.

	Coefficients	95% CI	*p*‐Value
**HOS**			
Age (years)	−0.32	[−0.51; −0.13]	<0.001
Sex (F/M)	2.97	[−0.85; 6.78]	0.126
BMI (kg/m^2^)	−0.23	[−0.73; 0.28]	0.375
Acetabular labral tear	−3.09	[−9.14; 2.96]	0.313
Femoral chondropathy	−2.24	[−5.92; 1.45]	0.231
Acetabular chondropathy	−1.85	[−5.81; 2.10]	0.355
Focal cartilage lesion (grade IV)	−1.28	[−5.30; 2.73]	0.527
**HOS‐SS**			
Age (years)	−0.25	[−0.49; −0.02]	0.037
Sex (F/M)	1.88	[−2.82; 6.59]	0.428
BMI (kg/m^2^)	0.15	[−0.47; 0.77]	0.636
Acetabular labral tear	−3.58	[−10.97; 3.82]	0.339
Femoral chondropathy	0.61	[−3.93; 5.14]	0.790
Acetabular chondropathy	−2.59	[−7.41; 2.24]	0.290
Focal cartilage lesion (grade IV)	−2.71	[−7.59; 2.17]	0.273
**HHS**			
Age (years)	−0.23	[−0.38; −0.08]	0.004
Sex (F/M)	2.18	[−0.92; 5.28]	0.166
BMI (kg/m^2^)	−0.24	[−0.64; 0.17]	0.255
Acetabular labral tear	−4.28	[−9.13; 0.57]	0.083
Femoral chondropathy	0.89	[−2.12; 3.89]	0.558
Acetabular chondropathy	−0.23	[−3.45; 3.00]	0.890
Focal cartilage lesion (grade IV)	−0.60	[−3.86; 2.66]	0.715
**WOMAC**			
Age (years)	0.16	[0.03; 0.28]	0.013
Sex (F/M)	−1.70	[−4.21; 0.80]	0.180
BMI (kg/m^2^)	0.12	[−0.21; 0.45]	0.482
Acetabular labral tear	2.16	[−1.80; 6.12]	0.281
Femoral chondropathy	−0.09	[−2.52; 2.35]	0.944
Acetabular chondropathy	0.03	[−2.57; 2.63]	0.981
Focal cartilage lesion (grade IV)	0.55	[−2.08; 3.18]	0.681

*Note*: Data were presented as coefficients.

Abbreviations: CI, confidence interval; HHS, Harris Hip Score; HOS, Hip Outcome Score; HOS‐SS, Hip Outcome Score – Sports Specific; WOMAC, Western Ontario and McMaster Universities Osteoarthritis Index.

The multivariate regression analysis showed that age was the only factor significantly associated with post‐operative HHS scores (*p* = 0.005). Specifically, for each additional year of age, the HHS score decreased by 0.22 points, indicating age‐related functional decline. The presence of an acetabular labral tear showed a trend toward lower HHS scores (−3.88 points), but this did not reach statistical significance (*p* = 0.102) (Table 4).

**Table 4:** Multivariate regression analysis for postoperative HHS.

HHS			
	Coefficients	95% CI	*p*‐Value
Age (years)	−0.22	[−0.37; −0.07]	0.005
Acetabular labral tear	−3.88	[−8.55; 0.79]	0.102

*Note*: Data were presented as coefficients. Factors were included in the multivariate analysis if with *p* < 0.1 at the univariate analysis.

Abbreviations: CI, confidence interval; HHS, Harris Hip Score.

## DISCUSSION

In the hip arthroscopy literature, articular cartilage health, particularly acetabular chondral damage, has been broadly reported as a significant predictor of postoperative outcomes. Several cohort studies have demonstrated that the presence and severity of acetabular cartilage lesions are associated with inferior patient‐reported outcomes and reduced likelihood of achieving clinically meaningful thresholds at mid‐term follow‐up. For example, Carreira et al. found that both low‐grade and high‐grade acetabular chondral lesions negatively predicted achievement of MCID and SCB for iHOT‐12 scores at a minimum 2‐year follow‐up, even after adjustment for age, BMI, gender and preoperative outcome scores [5]. Similarly, Lu et al. reported inferior outcomes after hip arthroscopy in patients with more advanced intraoperative cartilage lesions, supporting the traditional view that cartilage integrity influences postoperative recovery [15]. These findings align with broader clinical observations that focal cartilage pathology may contribute to residual symptoms and functional limitations following femoroacetabular impingement treatment.

In addition, long‐term cohort data indicate that combined femoral and acetabular chondral damage is associated with a higher risk of conversion to THA. In a 20‐year follow‐up study, age ≥40 years and the co‐existence of severe femoral and acetabular cartilage injury were associated with significantly greater odds of THA conversion, underscoring how cartilage pathology influences joint‐preserving outcomes in the long term [9]. Furthermore, multivariate analyses of large case series have demonstrated that older age is itself associated with more severe acetabular chondral damage and that higher grade cartilage lesions are more frequently observed in older patients undergoing hip arthroscopy [20].

However, the concept that cartilage status is the primary determinant of outcome is not universally supported. Age has also been shown to be a primary risk factor for severe cartilage damage at the time of hip arthroscopy, with nearly double the odds of severe acetabular damage at 45 versus 20 years of age [21].

Several large prospective cohorts and registry‐based analyses highlight that age independently predicts postoperative outcomes across a range of functional scores, sometimes with equal or greater effect size than localised cartilage grading. In the largest prospective series of >1000 patients, age along with preoperative PROMs, duration of symptoms, BMI and revision status remained a predictive factor for 2‐year postoperative outcomes in multivariate models [6]. Similarly, propensity‐matched comparisons between older and younger patient cohorts have shown that although improvement occurs in both groups, older patients may achieve lower absolute functional scores and higher rates of THA conversion, even in the absence of severe radiographic arthritis at surgery [7, 12].

Some large cohort studies have reported age as a significant independent predictor of postoperative functional outcomes after hip arthroscopy, emphasising the biological relevance of patient age in outcomes modelling [8].

From a clinical perspective, both patient age and intraoperative cartilage status are frequently considered when defining surgical indications for hip arthroscopy. Therefore, evaluating their relative contribution to postoperative functional outcomes remains clinically relevant, particularly at mid‐term follow‐up.

In the present study, patient age emerged as the only variable independently associated with postoperative functional outcomes in multivariate analysis, whereas femoral and acetabular cartilage severity did not retain statistical significance. One plausible explanation is that age represents a surrogate integrative marker of global joint health, encompassing not only localised cartilage lesions but also subclinical degenerative changes, altered tissue healing potential and other systemic factors not fully captured by intra‐operative cartilage grading. Another consideration is that the distribution of cartilage lesion severity within our cohort may have been relatively narrow, reducing the power to detect differential effects. Taken together, our findings suggest that while cartilage lesions remain clinically relevant, chronological age should be recognised as a key determinant of postoperative function and not merely a secondary consideration.

Importantly, the absence of an independent association between cartilage severity and postoperative outcomes in this cohort does not imply that cartilage integrity lacks clinical relevance. Rather, within the distribution of cartilage damage observed in our sample, its prognostic contribution may have been attenuated or partially mediated by age‐related factors. In addition, the generally high postoperative PROMs may have reduced the sensitivity to detect subtle differences between subgroups, introducing a potential ceiling effect.

These results highlight the importance of holistic preoperative evaluation and patient counselling. Rather than relying solely on chondral grading to guide surgical decision‐making, clinicians should incorporate age and global joint characteristics when discussing prognosis and expectations with patients undergoing hip arthroscopy. Recent studies have further emphasised the multifactorial nature of outcomes after hip‐preserving surgery, showing that capsular management, cam correction, patient‐specific predictive models and acetabular morphology, particularly in borderline dysplastic hips, may all influence postoperative results [1, 14, 19, 23]. Future research should aim to integrate advanced imaging, biochemical biomarkers and comprehensive morphological assessments to better delineate the relative contributions of cartilage health and biological age to long‐term joint‐preserving outcomes.

This study has several limitations. Its retrospective design may introduce selection bias, and all procedures were performed by a single surgeon, potentially limiting generalisability.

Although only four patients underwent bilateral procedures, hips were analysed as independent observations, and no adjustment for clustering was performed. While the small number of bilateral cases is unlikely to have substantially influenced the findings, this should be acknowledged as a methodological consideration.

Preoperative radiographic parameters were collected but were not included in the regression models. Therefore, the independent contribution of radiographic structural degeneration relative to age cannot be fully determined. Future investigations should incorporate integrated radiographic and intraoperative variables within predictive models.

Intraoperative cartilage status was categorised into broad severity groups, which may have reduced sensitivity in detecting more subtle associations with functional outcomes.

In addition, the a priori power analysis was based on the primary preoperative‐to‐postoperative comparison of the Hip Outcome Score and was not specifically designed to establish adequate power for multivariable regression analysis. Therefore, the multivariable findings should be interpreted with appropriate caution.

Finally, although age was independently associated with postoperative Harris Hip Score, the magnitude of the effect was limited and should be interpreted in the context of overall clinical decision‐making.

## CONCLUSION

In conclusion, patient age was independently associated with mid‐term functional outcomes following hip arthroscopy, whereas intraoperative cartilage status did not demonstrate an independent association within the limits of the present analysis. Although the magnitude of this effect was limited, age should be considered within a multifactorial framework when defining surgical indications and counselling patients. Cartilage integrity remains clinically relevant; however, its prognostic contribution may be influenced by age‐related factors and the overall joint environment. Further studies integrating radiographic and biological parameters are warranted to refine patient selection and optimise outcomes after hip arthroscopy.

## AUTHOR CONTRIBUTIONS

Andrea Burla conceptualise the idea of the study. Andrea Burla, Irene Tampieri, Valentina Fantoni, Francesco Aparo and Luca Vigliaroli collected data. Andrea Burla wrote the main manuscript text. Margherita Bonaiuti and Eleonora Olivotto were in charge of data evaluation and statistical analysis. Enrico Tassinari and Stefano Zaffagnini reviewed the article and supported the team during all phases of work.

## FUNDING

The authors have no funding to report.

## CONFLICT OF INTEREST STATEMENT

The authors declare no conflicts of interest.

## ETHICS STATEMENT

This study has been approved by the Local Ethical Committee of Rizzoli Orthopaedic Institute, Bologna,Italy (Protocol No. 0012125). All patients signed informed consent statement in order to participate in this study. Informed consent was obtained from all individual participants included in the study.

## Supporting information

STROBE Statement.

## Data Availability

The data that support the findings of this study are available from the corresponding author upon reasonable request.
