# Supplementary material for: Age and intraoperative cartilage status as determinants of mid‐term functional outcomes after hip arthroscopy
Source: J Exp Orthop. 2026 Jun 15;13(2):e70806. doi: 10.1002/jeo2.70806 (PMC13266902; doi:10.1002/jeo2.70806)
Supplement: Supplementary file 1 — STROBE Statement. [file JEO2-13-e70806-s001.docx]

STROBE Statement—Checklist of items in reports of ***cohort studies***

| **Item No.** | **Recommendation** | **Section in Manuscript** |
| --- | --- | --- |
| **Title and abstract** |  |  |
| 1 | Indicate the study design in the title or abstract | Title / Abstract |
| **Introduction** |  |  |
| 2 | Explain the scientific background and rationale | Introduction |
| 3 | State specific objectives and hypotheses | End of Introduction |
| **Methods** |  |  |
| 4 | Present key elements of study design early in the paper | Methods – Study Design |
| 5 | Describe the setting, locations, and relevant dates | Methods – Study Population |
| 6 | Eligibility criteria and selection of participants | Methods – Inclusion and Exclusion Criteria |
| 7 | Define outcomes, predictors, confounders | Methods – Outcome Measures |
| 8 | Data sources and measurement methods | Methods – Data Collection |
| 9 | Describe efforts to address potential bias | Methods / Limitations |
| 10 | Explain how the study size was determined | Methods – Statistical Analysis |
| 11 | Explain how quantitative variables were handled | Methods – Statistical Analysis |
| 12 | Describe statistical methods used | Methods – Statistical Analysis |
| **Results** |  |  |
| 13 | Report numbers of individuals at each stage of study | Results – Study Population |
| 14 | Give characteristics of study participants | Results – Table 1 |
| 15 | Report outcome events or summary measures | Results |
| 16 | Give unadjusted and adjusted estimates | Results – Regression Analysis |
| 17 | Report other analyses if performed | Results |
| **Discussion** |  |  |
| 18 | Summarise key results | Discussion |
| 19 | Discuss study limitations | End of Discussion |
| 20 | Interpretation of results | Discussion |
| 21 | Discuss generalisability | Discussion |
| **Other information** |  |  |
| 22 | Give the source of funding | Title Page / Funding Statement |
